# Response to Ng et al. on “Stimulus‐induced arousal with transient electroencephalographic improvement distinguishes nonictal from ictal generalized periodic discharges”

**DOI:** 10.1002/epi.70411

**Published:** 2026-08-12

**Authors:** Philippe Gélisse, William O. Tatum, Arielle Crespel, Peter W. Kaplan

**Affiliations:** ^1^ Epilepsy Unit Hôpital Gui de Chauliac Montpellier France; ^2^ Research Unit (Unité de Recherche sur les Comportements et Mouvements Anormaux) Institut national de la santé et de la recherche médicale (INSERM), U661 Montpellier France; ^3^ Department of Neurology Mayo Clinic College of Medicine and Health Sciences, Mayo Clinic Jacksonville Florida USA; ^4^ Epilepsy and EEG Unit Johns Hopkins Bayview Medical Center Baltimore Maryland USA

**Keywords:** electroencephalogram, nonconvulsive status epilepticus, periodic patterns, reactivity, triphasic waves

As the century began, Niedermeyer and Ribeiro^1^ cautioned against broad application of the emerging concept of nonconvulsive status epilepticus (NCSE). They stressed that true NCSE was rare. Moreover, many cases classified as NCSE in patients who were critically ill did not fulfill the criteria.^1^ They illustrated this distinction graphically, separating “true NCSE” from “false NCSE.” Absence status epilepticus (ASE) and NCSE in critically ill patients have very different clinical profiles. Patients with ASE are usually able to walk into the clinic, and unlike NCSE in critically ill patients, patients with ASE rarely require intensive care unit (ICU) admission (true NCSE). The reactivity to various forms of stimulation in our article was used as a bedside method to help the clinician identify nonictal patterns in their patients where a question of NCSE was present.^2^ Niedermeyer's “true” NCSE is the best example of NCSE, which is characterized by unreactive electroencephalographic (EEG) activity.

The framework proposed by Ng et al. contains three principal elements: first, a neutral or descriptive characterization of generalized periodic discharges (GPDs); second, classification of those patterns as ictal or nonictal according to the patient's response to a trial of antiseizure medication (ASM); and third, the ultimate diagnosis of NCSE based on the pharmacologic response.^3^ Yet exposing every patient who is suspected of manifesting NCSE to trials of ASM raises potential safety concerns. This concern is heightened in the elderly and across other vulnerable populations. Due to various disorders that can cause NCSE, many patients have ages at onset clustering in the mid‐60s^4^ to the early‐to‐mid 70s.^5^, ^6^ Intravenous benzodiazepines (BZPs) may cause marked sedation, and loading doses of other antiseizure agents can be difficult for frail patients to tolerate. Valproate, when administered as a loading dose, carries a risk of hyperammonemia.^7^ Phenytoin loading in elderly patients should be approached with caution due to an increased risk of cardiovascular instability, including hypotension and arrhythmias. BZDs or phenobarbital—even a single dose—can worsen hepatic encephalopathy, with a risk of prolonged stupor or coma.

Ng et al. argue that our article rests on problematic assumptions and imply “the cause of GPDs can be identified with sufficient reliability on EEG alone.” In this article, we sought to clarify that the underlying “cause” when addressed by reactivity may aid clinicians in differentiating ictal from nonictal patterns on EEG. GPDs with triphasic morphology are nonspecific patterns that may be encountered in acute encephalopathies, including postanoxia. However, they are also observed in basal meningitis, in Creutzfeldt–Jakob disease (CJD; Figure 1), and in severe brain injury (nonmetabolic triphasic waves [TWs]).^8^, ^9^ We do agree with Ng et al. that multiple factors may coexist in a single patient, complicating attribution of EEG abnormalities to a single underlying cause. For example, an elderly individual with chronic renal impairment and white matter disease who is hospitalized for pneumonia and treated with high‐dose penicillin may develop a multifactorial encephalopathy reflecting combined metabolic, toxic, septic, and hypoxemic mechanisms that are associated with GPDs.

**FIGURE 1:**
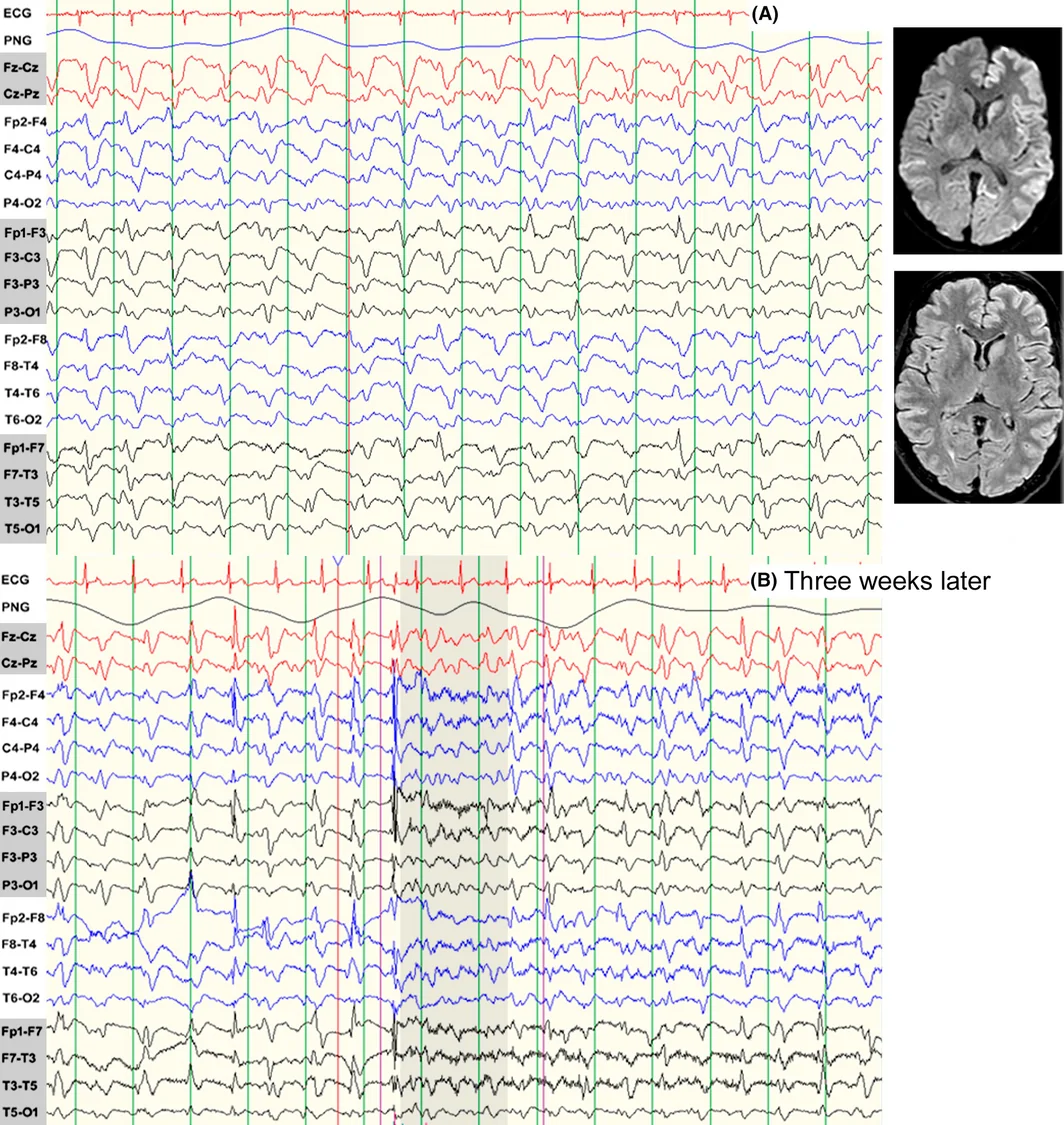
Forty‐nine‐year‐old person with rapidly progressive cognitive decline. Clinical features included ideomotor apraxia, dyscalculia, impaired attention, disorientation, bradyphrenia, and right‐hand dystonia, without aphasia or ataxia. Initial magnetic resonance imaging (MRI) showed cortical hyperintensity, predominantly in the right hemisphere. The first electroencephalogram (EEG) demonstrates nearly continuous irregular slow‐wave activity mixed with theta waves and occasional triphasic waves, reactive to nociceptive stimulation, raising suspicion for Creutzfeldt–Jakob disease (CJD). (A) Day 15 after admission. EEG shows continuous generalized periodic discharges (GPDs) with triphasic morphology. MRI was consistent with CJD, with cortical hyperintensity and involvement of the left caudate nucleus. (B) Three weeks later. EEG shows evolution toward periodic sharp wave complexes. EEG reactivity to auditory stimulation persists (gray area). The combination of clinical presentation, MRI findings, and GPDs with spiky morphologies of the periodic sharp wave complexes support the diagnosis of CJD. Preserved EEG reactivity, along with the clinical and electrophysiological pattern, argue against nonconvulsive status epilepticus. We thank Dr. Cassarini (Perpignan, France) for providing the case.

EEG findings containing GPDs must be interpreted within the context of historical, clinical, underlying biological, and neuroimaging considerations. The same concerns apply to treatment response. This may be misleading when it is based on an incorrect choice of ASM or improper dosing. BZPs when used as a challenge to distinguish an ongoing ictal EEG pattern primarily function as hypnotic agents. Their induction of sleep suppresses characteristic EEG patterns including TWs in metabolic or toxic encephalopathies and periodic sharp‐wave complexes in CJD, patterns that are otherwise present during fluctuating levels of wakefulness.^10^, ^11^ The effects of BZPs in hepatic encephalopathy and CJDs were well documented by 20th‐century authors,^10^, ^11^ yet these findings have received limited attention. Early 21st‐century reports of NCSE often relied solely on EEG improvement without clinical improvement following BZP administration. Improvement of the EEG pattern would be complicated by patients becoming sedated due to BZP medication. As a result, it was difficult to determine with certainty whether NCSE was present in such cases. Therefore, the Salzburg criteria introduced the category of “possible” NCSE.^12^

It was not until 2023 that a therapeutic alternative to BZPs really emerged. These agents involved the use of nonhypnotic medications. These were usually administered at two thirds or more of the standard loading dose used in patients with convulsive status epilepticus. However, older agents such as intravenous phenobarbital or (fos)phenytoin may still be used in developing countries where these drugs are more likely to be available.^13^ These protocols distinguish clearly between EEG response and clinical recovery, highlighting that EEG improvement is typically expected within minutes to an hour, depending on the drug, whereas clinical recovery may be delayed by up to 24 h after ASM administration.^13^ This contrasts with typical ASE (true NCSE), where BZP are used with a clear therapeutic intent rather than as a diagnostic test.^14^ In these cases, recovery of consciousness is parallel to the cessation of generalized spike‐and‐wave activity.

Taking a universal and singular approach to GPDs goes against the principles of precision medicine, for example, the classical pattern of asymmetric periodic sharp wave complexes in CJD that may evolve into GPDs. This is a condition that does not warrant a therapeutic trial. This pattern is well known to be associated with CJD and within the appropriate context of the recording, strongly supports the clinical diagnosis (Figure 1). Intravenous amphetamine has occasionally been administered in CJD with periodic sharp wave complexes, producing brief improvements in both consciousness and EEG.^15^, ^16^ These changes are used as an important practical benchmark when assessing responsiveness to ASMs. However, amphetamines have no antiseizure effects. No published data support their use specifically in patients with metabolic encephalopathies with TWs. In CJD, chloral hydrate has also been observed to improve EEG findings, like BZPs.^17^ Nonetheless, choral hydrate has no antiseizure activity and like BZPs may induce sedation and sleep cycling impacting GPDs in CJD. Recognizing typical TWs and avoiding conclusions based solely on EEG improvement after a drug challenge helps prevent unnecessary interventions. It also ensures that antiseizure testing is reserved for situations in which it is truly warranted to minimize drug‐induced complicating factors.

One criticism, according to Ng et al., is that we suggested that “ictal GPDs or NCSE can be distinguished from non‐ictal GPDs or non‐NCSE based solely on EEG findings.” The idea that NCSE can be diagnosed with a high degree of certainty from GPDs when relying purely on reactivity was not the conclusion drawn from our earlier work.^8^, ^18^ We do agree that GPDs with triphasic morphology—long considered suggestive of metabolic encephalopathy—do not reliably exclude NCSE on EEG grounds alone. Even in cases where typical TWs are present, patients may still also have electrographic or electroclinical seizures. An older study (1976) suggested that approximately one third of patients with acute renal failure experienced convulsive seizures.^19^ However, since that study was conducted, advances in critical care management including continuous EEG monitoring have significantly improved patient outcomes.

As previously mentioned, the clinical context remains indispensable, along with semiological features that may be indicative of seizures or NCSE including ocular deviation, subtle clonic jerking, and other stereotyped behaviors. An abrupt onset points more strongly toward NCSE, whereas uniform GPDs < 2.5 Hz that lie on the ictal–interictal continuum, in association with a progressive decline in alertness and some degree of preserved reactivity or awareness, tend to indicate a metabolic or multifactorial cause. In contrast, GPDs that are not strictly periodic and display an evolving pattern (increasing frequency, amplitude, or spatial spread),^20^ GPDs associated with neurological signs, and GPDs occurring in the context of acute brain injury are highly suggestive of NCSE. In these situations, the nonreactivity of the GPDs favors its diagnosis. At the same time, clinicians should avoid uncritically labeling multifocal myoclonus associated with acute renal failure or toxic encephalopathies as epileptic in origin in need of ASM. Various abnormal movements frequently occur in the ICU, including tremors and asterixis.

Following severe cerebral insults such as trauma or hemorrhage, failure to demonstrate neurological recovery despite discontinuing sedative drugs may add clinical support to interpret GPDs as NCSE. However, TWs may be seen during sedation withdrawal, especially after iatrogenic burst–suppression with pentobarbital or high‐dose propofol. This pattern does not represent a reactivation of NCSE in comatose patients. Rather, it is a well‐described and expected post‐burst–suppression pattern recognized since the 1960s, in the clinical context of drug overdoses from short‐acting barbiturates (secobarbital, pentobarbital) used as hypnotics. In this setting, the appropriate therapeutic approach is to continue sedation withdrawal, as these TWs reflect a transient, nonepileptic phenomenon that resolves with awakening (Figure 2).^11^, ^21^

**FIGURE 2:**
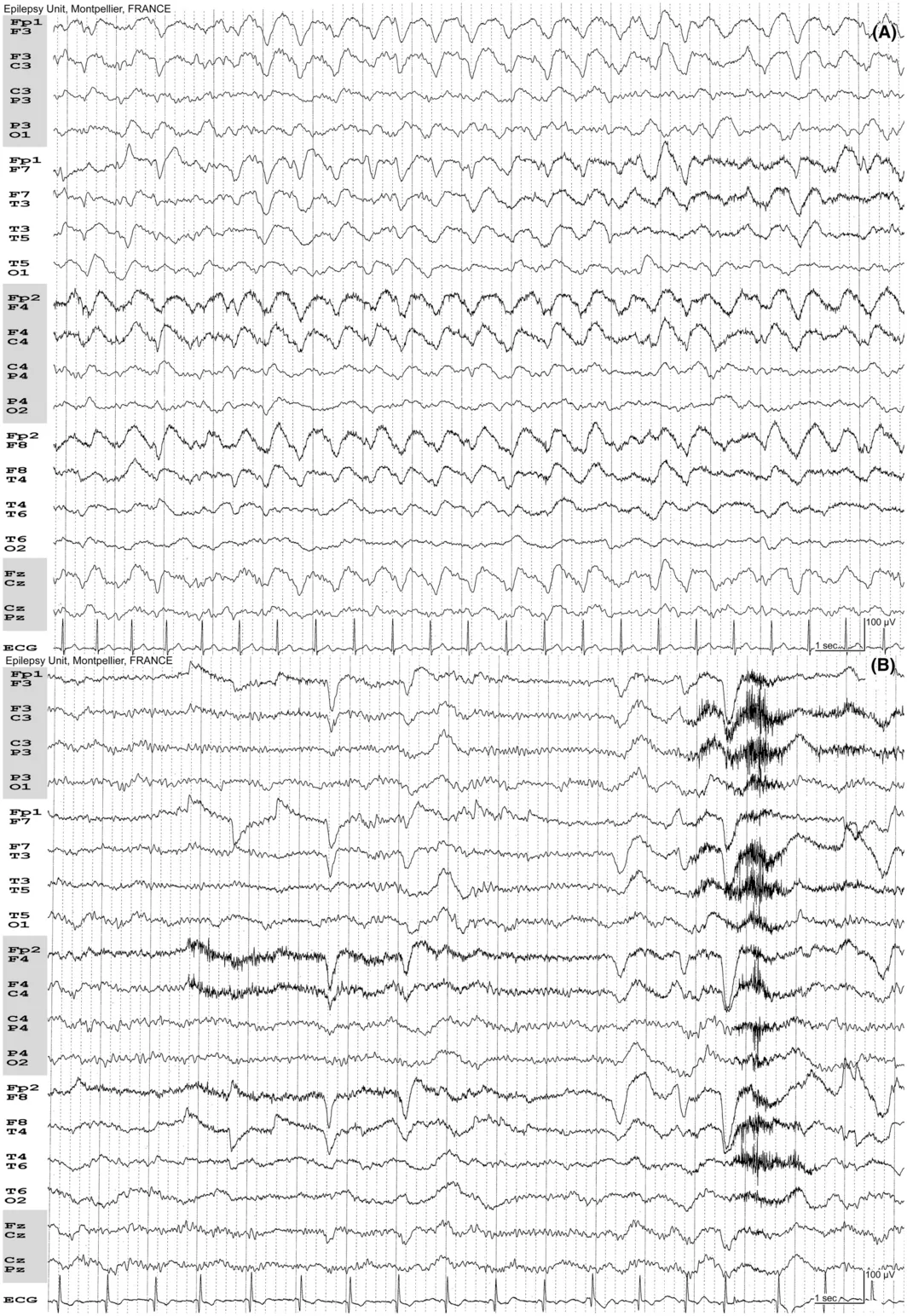
(A) A patient in their 70s was admitted to the intensive care unit for general anesthesia following generalized convulsive status epilepticus caused by a cerebral hemorrhage. The patient was also treated with intravenous levetiracetam. During sedation weaning, electroencephalogram (EEG) showed diffuse, nearly continuous triphasic waves. This pattern is consistent with a transitional state between propofol‐induced coma and full awakening and does not warrant resumption of sedation or escalation of antiseizure therapy. (B) Four days later, the patient awoke, and the EEG normalized. Artifacts from lateral eye movements, eyelid blinking, and muscle activity are now present.

Neuroimaging technologies are advancing at an exponential pace. Magnetic resonance imaging (MRI) with arterial spin labeling sequences and computed tomography (CT) perfusion now enable relatively straightforward detection of focal SE.^22^, ^23^, ^24^ However, neuroimaging remains of limited utility in cases of suspected generalized NCSE. To better define the imaging correlates of generalized nonconvulsive seizures, future research should aim to capture generalized seizure activity directly during imaging, whether incidentally (e.g., absence seizures occurring during acquisition) or through prospective studies that systematically evaluate patients with GPDs and ASE.

MRI may now reveal characteristic findings in hepatic or other toxic–metabolic encephalopathies,^25^ and although such findings can offer valuable supportive information, they are insufficient to reliably exclude SE. In this context, the detection of focal seizure activity on MRI or CT perfusion may prove particularly informative, potentially guiding clinical decision‐making when EEG remains ambiguous or when continuous EEG monitoring is not available.

To conclude, we would like to thank Ng et al. for their perspective, which has helped stimulate discussion surrounding this important point. The aim of our article was to define a bedside clue of reactivity to use as an adjunct to electroclinical decision‐making by addressing GPDs as a potentially ictal EEG pattern. It was not proposed as part of a formal definition for NCSE already established by the International League Against Epilepsy. GPDs with triphasic morphology may, in some cases, represent a pattern of NCSE. Toxic–metabolic or multifactorial encephalopathies can be associated with seizures and may need urgent ASMs. We do not consider toxic–metabolic encephalopathy with TWs and SE to be mutually exclusive; they may coexist in the same patient. Because SE is a self‐sustaining process with impaired spontaneous termination, external stimulation—or even spontaneous patient movement—is not likely to be interrupted with alerting stimuli. EEG reactivity assessment is simple, straightforward, safe, and can be used at the bedside.^26^, ^27^, ^28^

## AUTHOR CONTRIBUTIONS

**Philippe Gélisse:** Conceptualization; writing—original draft; writing—review and editing. **William O. Tatum:** Writing—review and editing. **Arielle Crespel:** Writing–review and editing. **Peter W. Kaplan:** Writing—review and editing.

## FUNDING INFORMATION

This study received no specific funding.

## CONFLICT OF INTEREST STATEMENT

P.G. has received support for teaching programs from UCB and Eisai and royalties for publishing from John Libbey Eurotext. W.O.T. has received a stipend from Elsevier as Editor‐in‐Chief of *Epilepsy Behavior Report*. He serves on the editorial board for the *Journal of Clinical Neurophysiology* and *Epilepsy Behavior Reports*. He is a consultant and the national medical director for Synergy Medical Solutions. He holds patents or patents pending for intraoperative monitoring devices (#62527896, #62770362). He has received royalties for publishing from Demos Publishers, Springer Publishing, Wiley, Cambridge and MedLink and honoraria for speaking engagements from the American Academy of Neurology, American Epilepsy Society, and American Clinical Neurophysiology Society. He has received research support from Mayo Clinic, Martin Family Foundation, UCB, Neurelis, LivaNova, Minder, Xenon Pharma, and McElvey Fund. A.C. has received support for teaching programs from UCB and Eisai and royalties for publishing from John Libbey Eurotext. She was an advisory board member for Eisai‐France. P.W.K. has been on the board of the American Board of Clinical Neurophysiology and the American Clinical Neurophysiology Society and has consulted for Ceribell. We confirm that we have read the Journal's position on issues involved in ethical publication and affirm that this report is consistent with those guidelines.

## ETHICS STATEMENT

This study analyzed routine EEG data collected as part of standard clinical care. The data were fully anonymized (including only age, disease, and EEG findings), and no identifiable patient information was used, so formal ethical committee approval and informed consent were not required, and the study complies with institutional/national guidelines for retrospective analyses of anonymized clinical data.

## Data Availability

The data that support the findings of this study are available from the corresponding author.
